# Different Sources of Copper Effect on Intestinal Epithelial Cell: Toxicity, Oxidative Stress, and Metabolism

**DOI:** 10.3390/metabo10010011

**Published:** 2019-12-23

**Authors:** Runxian Li, Yang Wen, Gang Lin, Chengzhen Meng, Pingli He, Fenglai Wang

**Affiliations:** 1 State Key Laboratory of Animal Nutrition, College of Animal Science and Technology, China Agricultural University, Beijing 100193, China; lrx1024@cau.edu.cn (R.L.); weny@cau.edu.cn (Y.W.); SY20183040640@cau.edu.cn (C.M.); 2 Institute of Quality Standards and Testing Technology for Agricultural Products, Chinese Academy of Agricultural Science, Key Laboratory of Agrifood Safety and Quality, Ministry of Agriculture, Beijing 100081, China; lingang@caas.cn

**Keywords:** copper, cytotoxicity, bioavailability, oxidative stress, intestinal epithelial cell

## Abstract

Copper (Cu) is widely used in the swine industry to improve the growth performance of pigs. However, high doses of copper will induce cell damage and toxicity. The aim of this study was to evaluate toxicity, bioavailability, and effects on metabolic processes of varying copper sources using porcine intestinal epithelial cells (IPEC-J2) as a model. The IPEC-J2 were treated with two doses (30 and 120 μM) of CuSO_4_, Cu Glycine (Cu-Gly), and Cu proteinate (Cu-Pro) for 10 h, respectively. Cell damage and cellular copper metabolism were measured by the changes in cell viability, copper uptake, oxidative stress biomarkers, and gene/protein expression levels. The results showed that cell viability and ratio of reduced and oxidized glutathione (GSH/GSSG) decreased significantly in all treatment groups; intracellular copper content increased significantly in all treatment groups; total superoxide dismutase (SOD) activity increased significantly in the 120 μM exposed groups; SOD1 protein expression levels were significantly upregulated in 30 μM Cu-Pro, 120 μM Cu-Gly, and 120 μM Cu-Pro treatment groups; intracellular reactive oxygen species (ROS) generation and malondialdehyde (MDA) content increased significantly in 30 μM treatment groups and 120 μM CuSO_4_ treatment group. *CTR1* and *ATP7A* gene expression were significantly downregulated in the 120 μM exposed groups. While upregulation of *ATOX1* expression was observed in the presence of 120 μM Cu-Gly and Cu-Pro. *ASCT2* gene expression was significantly upregulated after 120 μM Cu-Glycine and CuSO_4_ exposure, and *PepT1* gene expression was significantly upregulated after Cu-Pro exposure. In addition, CTR1 protein expression level decreased after 120 μM CuSO_4_ and Cu-Gly exposure. PepT1 protein expression level was only upregulated after 120 μM Cu-Pro exposure. These findings indicated that extra copper supplementation can induce intestinal epithelial cell injury, and different forms of copper may have differing effects on cell metabolism.

## 1. Introduction

Copper is an essential trace element in animals that is beneficial to hematopoiesis, growth, metabolism, and various important physiological activities of life [1]. For a long time, copper has been added to swine diets at high concentrations to reduce infection and promote growth, especially for weanling pigs [2,3]. However, high concentrations of copper in the diet may lead to organ damage of swine and pose a threat to the environment [4,5]. According to the latest regulations of China and the European Union, the maximum level of copper in piglets feed should not exceed 150 and 125 ppm, respectively [6,7]. Therefore, many researchers are exploring the appropriate concentration of copper fed to swine.

The traditional method of copper supplementation has been through using inorganic copper sources, such as CuSO_4_. However, inorganic coppers are poorly absorbed by swine, and the diets are often supplemented with more copper than they actually need. Huang et al. evaluated the effect of dietary copper amount on weanling pigs. Compared with control pigs (0.88 mg/L), soluble copper concentrations in the proximal jejunal (5.54 mg/L) were increased significantly when fed with a diet containing 225 mg of copper per kilogram [8]. Previous studies have reported that Cu overload leads to oxidative stress and subsequent oxidative damage to proteins, lipids, and nucleic acids because of its ability to generate free radical species, such as hydroxyl radicals (OH^−^) [9,10]. At the cellular level, copper ions in various oxidation states can participate in oxidation and reduction reactions. For example, the reduction of cupric ions forming cuprous ions by superoxide radical anions that may catalyze the formation of hydroxyl radicals via the Fenton reaction inducing oxidative stress and thereby cell injury [11]. Over the last two decades, organic copper sources, such as amino acid chelate and proteinate, have been increasingly used in livestock. Many studies have shown that organic sources of copper have higher bioavailability and lower mineral excretion than inorganic sources, with improved growth performance of pigs [12,13]. However, one of the major challenges when investigating high absorption of organic copper is that there is little known about the metabolism mechanisms in livestock.

Copper metabolism includes many processes related to copper uptake, intracellular distribution of copper, and copper export. In general, copper ions dissociate from their organic or inorganic carriers in the intestinal lumen and are absorbed via a passive or active route into enterocytes lining the gastrointestinal tract. After processing in the liver to bound albumin, copper is distributed to other tissues in a second phase, and excess copper is excreted in feces. Because both copper deficit and excess are deleterious to the organism [14], copper levels are tightly regulated by a complicated system, which has many vital proteins working together to ensure a stable level of copper in organisms. The high-affinity copper transporter 1 (CTR1) is the primary player in the uptake of extracellular copper. ATOX1 can carry copper to copper-ATPases (ATP7A and ATP7B) in the Golgi apparatus, and ATP7A and ATP7B are ATP-dependent translocators of copper across membranes when intracellular copper levels are high [15,16]. Studies have shown that organic metals, such as proteinate, may be absorbed attached to PepT1, a brush border membrane-bound di- and tri-peptide transporter, which has been identified in all major livestock species. This transporter is not overly specific, as molecules other than di- and tri-peptides can be transported through it. Joanne Keenan et al. compared organic and inorganic copper exposure to HT29 and Caco-2 cells. Their findings showed that PepT1, ZnT1, and CTR1 were increased, particularly for CuSO_4_ and copper proteinate in Caco-2 cells [17].

To the best of our knowledge, there is scarce study about different damage and metabolic mechanisms between organic and inorganic copper in pigs at the cellular level. In this study, we analyzed the effects of organic and inorganic copper sources on porcine small intestinal epithelial cell line (IPEC-J2) to evaluate their effect on cell growth, oxidative stress, copper absorption efficiency, and copper metabolism, to provide data support for the precise use of copper in swine production. Three sources of copper (copper sulfate—CuSO_4_, copper glycinate—Cu-Gly, and copper proteinate—Cu-Pro) were compared.

## 2. Materials and Methods

### 2.1. Reagents

Copper sulfate, copper bisglycinate, 2-Thiobarbituric acid (TBA), 5, 5′-Dithiobis (2-nitrobenzoic acid) (DTNB), sulfosalicylic acid, and propionaldehyde diethyl acetal were purchased from Sigma Company (St. Louis, MO, USA). Copper proteinate was obtained from a commercial premix company (Bioplex^®^, Alltech Inc., Nicholasville, KY, USA). Fetal bovine serum (FBS) and Dulbecco’s modified Eagle’s medium-F12 (DMEM-F12) was obtained from Hyclone (Logan, UT, USA). A BCA TM Protein Assay Kit was obtained from Pierce (Rockford, IL, USA). A Protein G agarose column was obtained from Upstate Biotechnology (Lake Placid, NY, USA). ELISA plates (96 wells) and other cell culture plastic wares were obtained from Costar (Cambridge, MA, USA). HPLC grade methanol and acetonitrile were obtained from Fisher Scientific International (Hampton, NH, USA). HPLC grade formic acid and ammonium acetate were purchased from Dima Technology (Richmond Hill, Canada). Water used was ultrapure water (Milli-Q, Millipore Corporation, Billerica, MA, USA).

### 2.2. Apparatus and Procedures

A Heraeus HERA Cell CO_2_ incubator (Kendro Lab., Asheville, NC) was used for cell cultures. A high-speed refrigerated centrifuge (Sigma 3–30 K, Sigma, Munich, Germany) was used for sample pretreatments. SDS-PAGE electrophoresis slot and Western blot electrophoresis transfer slot were purchased from BioRad Laboratories (Hertfordshire, England). A Desktop Constant Temperature Oscillator (Jing Hong, Shanghai, China) was used to promote sample extraction. MDA was analyzed by an Agilent 1200 HPLC (Agilent Technologies, Fermont, CA) system. GSH and GSSG were analyzed on an Agilent 1200 UHPLC system coupled with a 6460 Mass Selective Detector. Copper content was done by Agilent Technologies 7700 Series inductively coupled plasma-mass spectrometry (ICP-MS) system. The quality of RNA was determined using a spectrophotometer (NANODROP2000, Thermo Scientific, Waltham, MA, USA).

### 2.3. Cell Culture and Treatment

The Porcine small intestinal epithelial cell line (IPEC-J2) was obtained from the American Type Culture Collection. And cells were cultured in DMEM-F12 media (Hyclone) additionally supplemented with 10% FBS (Gibco), 1% ITS (Gibco), 50 U/mL of penicillin, 50 μg/mL of streptomycin (Gibco) and 1% HEPES (Gibco). Cells were seeded (2.0 × 10^5^ cells/mL) in 25 cm^2^ culture dishes in a 5% CO_2_ humidified incubator at 37 °C for 24 to 48 h, then cells were divided into several groups and cultured with complete medium containing 30 to 120 μM of copper sulfate (CuSO_4_), copper glycinate (Cu-Gly,) or copper proteinate (Cu-Pro), respectively. The exposure concentration was selected based on previous researches, which basically corresponds to the soluble copper concentrations in the proximal jejunal in vivo model [6]. The control group was incubated with complete medium with no extra copper added. After 10 h, the cells were collected for uptake and metabolomic analysis.

### 2.4. Cell Viability Assay

Cellular viability assays were conducted with a water-soluble tetrazolium salt-8 (WST-8) cell counting kit (CCK-8, Dojindo, Japan) according to the method described in the manufacturer’s instructions. IPEC-J2 in cell culture medium (90 μL/well) at a density of 1 × 10^4^ cells/well were added into the 96-well plate and then treated with varying concentrations of CuSO_4_, Cu-Gly, and Cu-Pro (10 μL/well) for 10 h exposure. Afterward, the CCK-8 assay reagent (10 μL/well) was added into the wells for 1 h incubation. Then, the absorbance of the dye was detected on a microplate reader (BIO-RAD 680, Hercules, CA, USA) at 450 nm.

### 2.5. EdU Retention Assay

After copper treatment, cells were incubated with culture medium containing 50 μM EdU (RiboBio, China) for 2 h at 37°C. Then cells were washed with PBS, fixed by 4% paraformaldehyde, and incubated with 2 mg/mL glycine. After permeabilized with 0.5% triton X-100, cells were incubated with Apollo staining solution for 30 min. Then, the DNA of the cells were stained with Hoechst for 30 min. Cells were observed and captured using a fluorescent microscope (EVOS M5000, Thermo Scientific, Waltham, MA, USA).

### 2.6. Determination of Copper Uptake

Cells were seeded in 25 cm^2^ culture dishes for 24 h and treated with different concentrations of CuSO_4_, Cu-Gly, and Cu-Pro for 10 h, then the cell culture medium was removed, and the cells were washed twice with PBS. After harvesting with trypsin and washing with PBS, the cells were digested by 98% HNO_3_ and Milli-Q water (V:V = 1:1) at 100 °C for about 1 h. Then after centrifugation (3000 rpm, 5 min), the supernatants were collected for the determination of copper uptake. The determination of copper in IPEC-J2 cells was done by ICP-MS according to the method described earlier [18].

### 2.7. Measurement of ROS

Intracellular reactive oxygen species(ROS) generation was determined by using 2, 7-dichlorofluorescin diacetate (DCFH-DA). Briefly, 1 × 10^4^ cells were cultured in a 96-well plate, after copper treatment, cells were washed and incubated with 200 μL DCFH-DA (10 μM) working solution at 37 °C for 30 min, then cells were washed 3 times with PBS. Afterward, the fluorescence intensities from every well were detected on a microplate reader (BioTek Synergy4, Winooski, VT, USA), excitation and emission wavelengths were 498 and 528 nm, respectively.

### 2.8. Measurement of GSH and GSSG

As described previously [19], an extraction and fast analytical method for simultaneous analyses of reduced and oxidized glutathione (GSH and GSSG, respectively) in cells were applied by LC-MS/MS to get more reliable results. Harvested cells were dissolved in 300 μL PBS, the samples were treated with 10 μL of 0.5 mM DTNB solution in methanol, mixed, and incubated at room temperature for 15 min, and the reaction was stopped by the addition of 60 μL of cold sulfosalicylic acid solution (20% *w*/*v*) to precipitate proteins. After 15 min at 4 °C, the precipitated proteins were removed by centrifugation (20,800× *g* at 4 °C for 15 min). The clear supernatant (150 μL) was used for LC-MS/MS analyses.

Chromatographic separation of the GSH and GSSG in cells was achieved on an Agilent Eclipse BEH 18 column (2.1 mm × 150 mm, 3.5 μm). The column temperature was 30 °C. The mobile phase consisted of solutions A (0.1% aqueous formic acid solution) and B (acetonitrile). A gradient program was used for elution: 5% solution B (initial), with 90% solution B (from 0 to 7 min), 90% solution B (from 7 to 10 min), and 95% solution B (from 10 to 10.1 min). A 5-min equilibration was necessary before the next injection. The mobile phase was delivered at a flow rate of 0.3 mL/min. The optimized electron spray ionization condition was gas temperature 350 °C, gas flow 5 L/min, sheath gas temperature 350 °C, sheath gas flow 7 L/min, and capillary voltage 3500 V. High-purity nitrogen was used as the nebulizing gas. Positive ions were monitored. Multiple reaction monitor mode was applied for quantitative and qualitative analysis. The ion transitions, *m*/*z* 613.2/482.4 and 613.2/355.1, were selected and used for quantification and identification of the GSSG, respectively. The ion transitions, *m*/*z* 308.3/179 and 308.3/162, were selected for quantification and identification of the GSH, respectively. The amounts of GSH and GSSG in the cells were quantified using a calibration curve.

### 2.9. Measurement of SOD Activity

As mentioned above, cells were treated and harvested for the detection of oxidative stress biomarkers. The total superoxide dismutase (SOD) activity was assessed using an assay kit (Nanjing Jiancheng Bioengineering Institute, China) according to the manufacturer’s instructions.

### 2.10. Measurement of MDA Content

MDA was measured by an acid reactive substance (TBARS) assay. In briefly, the TBARS assay was carried out by adding 1.0 mL of trichloroacetic acid (0.5 M) and l.0 mL TBA (0.02 M) to each tube that contained cells and boiling the mixture for 45 min. After cooling to room temperature and centrifugation (3000 rpm, 5 min), the concentration of MDA in the supernatant was detected by HPLC.

Chromatographic separation of MDA was achieved on a Waters Atlantis dC18 column (particle diameter 5 µm, 150 × 4.6 mm i.d.). The mobile phase was 10 mM ammonium acetate aqueous solution–methanol (70:30, *v*/*v*). The mobile phase flow rate was 1.0 mL/min, and the injection volume was 10 µL. Detection with a UV–vis detector was carried out at 532 nm. The amounts of MDA in samples were quantified using a calibration curve developed with the MDA standard solution.

### 2.11. Quantitative Real-Time Polymerase Chain Reaction (RT-qPCR)

Primers were designed with Primer 5.0 software using Sus scrofa sequences from the NCBI database and synthesized by Beijing Sunbiotech Co. Ltd. (Bejing, China). All primer sequences are listed in Table 1. Total RNA was isolated with RaPure Total RNA Kits (MAGEN Biology, Beijing, China) and reverse-transcribed with the PrimeScript™ RT reagent Kit (TAKARA, Tokyo, Japan). The quality of RNA was determined by calculating the OD 260/280 ratio. Real-time PCR was performed on a Roche Light Cycler 96 in a 10 μL reaction volume system, containing 0.3 μL of each primer, 5 μL TB Green™ Premix Ex Taq™ (TAKARA, Tokyo, Japan), 0.3 μL RNase free H_2_O, and 4 μL cDNA template. PCR cycling conditions were 94 °C, 5 min; 38 cycles each of 94 °C for 30 s; 59 °C for 30 s; 72 °C for 30 s; and a final extension at 72 °C for 10 min.

### 2.12. Western Blot Analysis

Samples (20 μg of total protein) were separated by 12% SDS-PAGE, followed by electrotransfer to PVDF membrane (GE Health Care). The membranes were blocked with 5% skim milk in TBS containing 0.05% Tween-20 (TBST) and incubated overnight at 4 °C with the antibodies rabbit anti-Ctr1 (1:1000), rabbit anti-PepT1 (1:1000), rabbit anti-SOD1 (1:1000), and rabbit anti-Actin (1:1000). After washing three times with TBST, the membranes were incubated with horseradish peroxidase (HRP) conjugated antibody (Beyotime) at 37 °C for 1 h. After washing three times with TBST, the membrane was detected using clarity-enhanced chemiluminescence (ECL) reagent (Thermo Fisher Scientific). Image J software was used to analyze the gray value of protein bands, and the gray value of target proteins and corresponding internal reference proteins were statistically analyzed.

### 2.13. Statistical Analysis

All statistical analyses were performed using SPSS 19.0 software, and values are presented as means ± standard error of means (SEM). Data were examined with two-way ANOVA by Tukey’s multiple comparisons test. The value of *p* < 0.05 was considered as statistically significant.

## 3. Results

### 3.1. The Effects of Copper on Cell Viability

**Figure 1:**
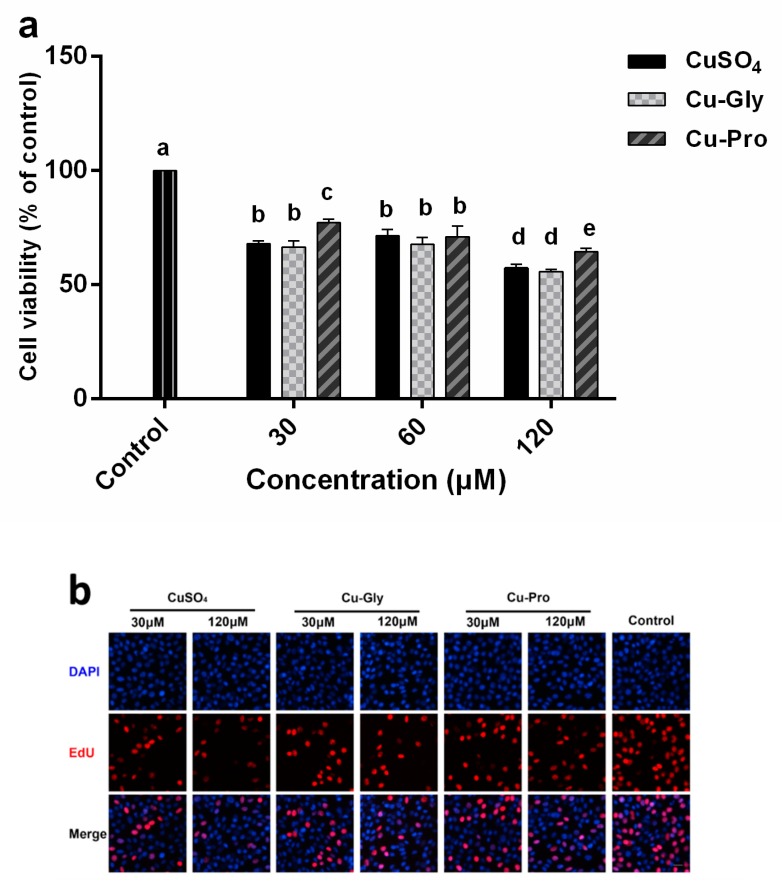

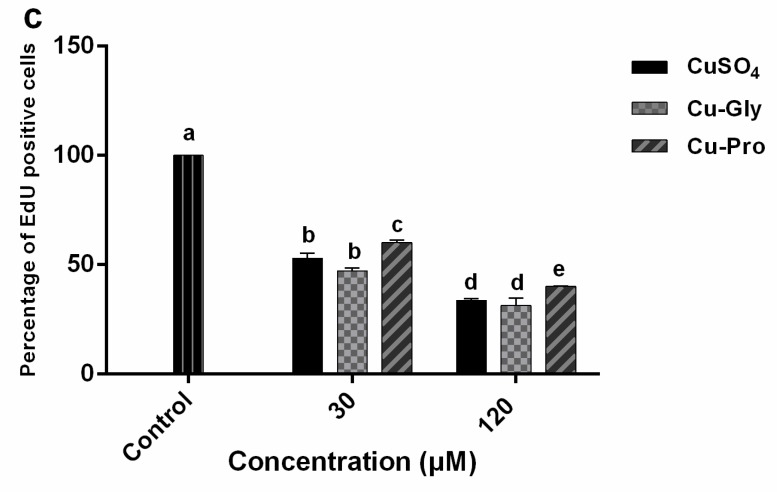
Cell viability in response to different copper concentrations for 10 h. (**a**) Cell counting kit 8 (CCK-8) cell viability assay. (**b**) EdU staining assay. (**c**) Statistical analysis of the percentage of EdU positive cells. The result was represented as a percentage of the control. Data represent mean values ± standard deviation (*n* = 4). Significant differences between processing groups are represented by different lowercase letters (*p* < 0.05).

First, the copper incubation time was optimized by the use of IPEC-J2 cells without adding copper. Cells continued to be cultured for 2, 6, 10, 14, 18, 24, and 36 h after reaching 100% confluence. There was no significant change in cell viability for 2 to 10 h of culture, and cell viability began to decline after 14 h through 36 h of culture. Thus, the optimal copper exposure time was determined to be 10 h. The viability of the IPEC-J2 cells after the incubations of different copper sources for 10 h can be seen in Figure 1a. Cell viability was significantly decreased after treatment with 30, 60, and 120 μM concentrations of CuSO_4_, Cu-Gly, and Cu-Pro compared to control. The cell viability after Cu-Pro incubation was significantly higher than the other two copper sources at 30 and 120 μM. As cells exposed to 30 and 60 μM copper showed similar cell viability profiles, 30 μM copper was chosen as dose1, and 120 μM was chosen as dose2 for subsequent experiments.

In addition, we used another method to evaluate cell proliferation, and the results of EdU staining are shown in Figure 1. As we can see in Figure 1b, EdU positive cells decreased with the increase in copper concentration in all copper treatment groups in comparison to the control group. Then, three captured fields were randomly selected in every group to calculate the percentage of EdU positive cells. Figure 1c shows that the percentage of EdU positive cells was significantly decreased after treatment with CuSO_4_, Cu-Gly, and Cu-Pro compared to control. In addition, the percentage of EdU positive cells after Cu-Pro incubation was significantly higher than the other two copper sources at both doses.

### 3.2. Copper Uptake in Cells

**Figure 2:**
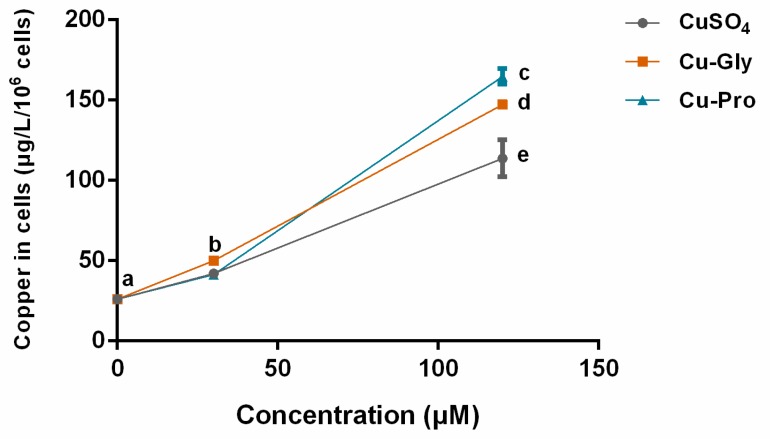
Intracellular copper concentration per 10^6^ cells. Cells were treated with CuSO_4_, Cu Glycine (Cu-Gly), and Cu proteinate (Cu-Pro) at the concentrations of 30 and 120 μM for 10 h. The result was measured by ICP-MS. Data represent mean values ± standard deviation (*n* = 3). Significant differences between processing groups are represented by different lowercase letters (*p* < 0.05).

Since the bioavailability of the inorganic and organic copper sources may lead to different uptake rates, the intracellular copper levels were measured by ICP-MS. After exposing IPEC-J2 to CuSO_4_, Cu-Gly, and Cu-Pro at the concentrations of 30 and 120 μM for 10 h, the Cu contents in cells were significantly increased with the increase in copper exposure compared to control, as shown in Figure 2. At 120 μM, significant differences in uptake rates among the three kinds of copper were observed. Compared to control, the highest copper absorption was in the Cu-Pro treated group with a 6.3-fold increase; the lowest was the CuSO_4_ treated group with a 4.4-fold.

### 3.3. The Effects of Copper on Oxidative Stress and Antioxidant Activity

**Figure 3:**
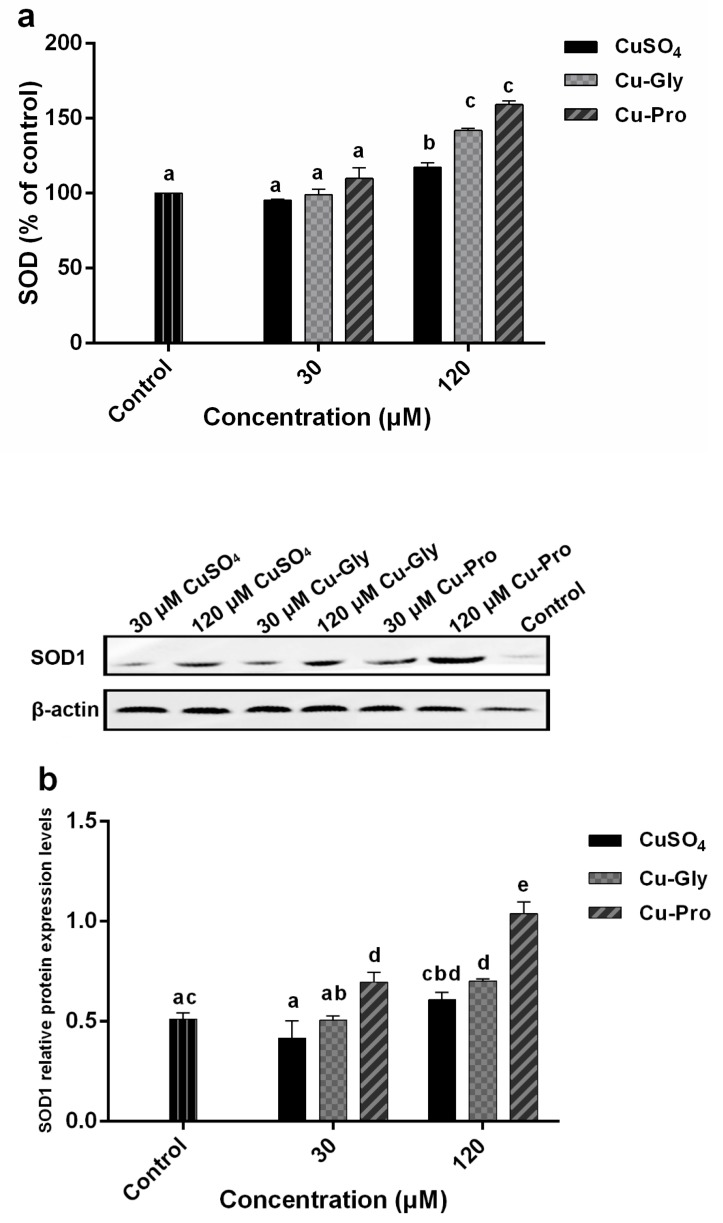

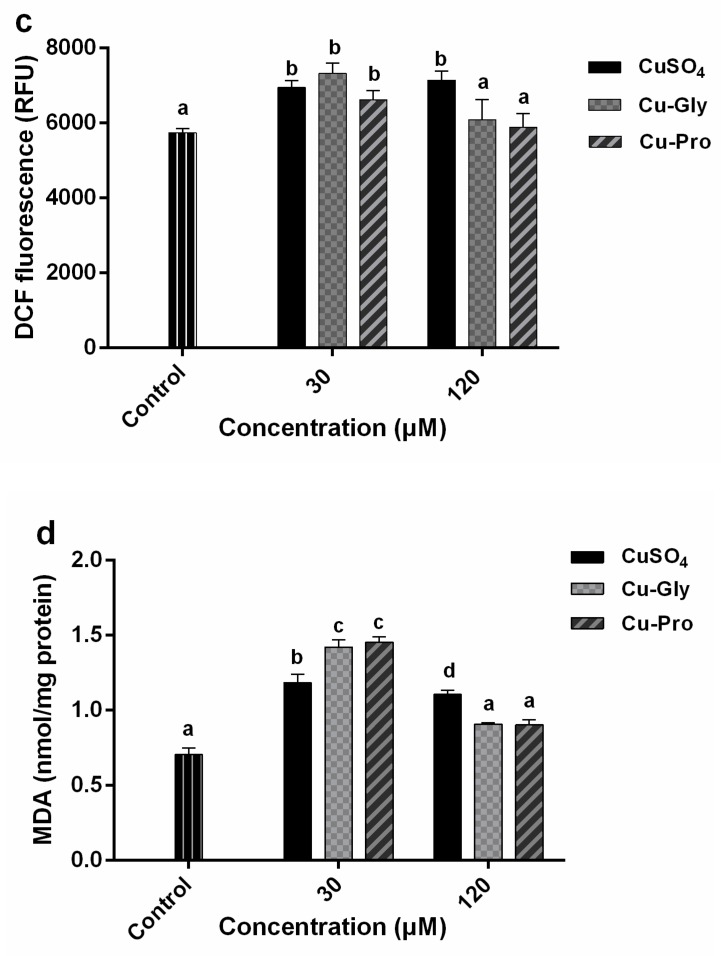
Copper induced oxidative stress in porcine small intestinal epithelial (IPEC-J2) cells. Cells were treated with CuSO_4_, Cu-Gly, and Cu-Pro at the concentrations of 30 and 120 μM for 10 h. (**a**) Total superoxide dismutase (SOD) activity was assessed using an assay kit. The result was expressed as a percentage of the control. (**b**) SOD1 protein expression levels were measured by Western blotting. The densitometry of blot images normalized to β-actin levels for each lane. (**c**) Reactive oxygen species (ROS) was determined by using 2, 7-dichlorofluorescin diacetate (DCFH-DA). (**d**) Malondialdehyde (MDA) was measured by an acid reactive substance (TBARS) assay. The result was corrected with the total protein content of cells. Data represent mean values ± standard deviation (*n* = 3). Significant differences between processing groups are represented by different lowercase letters (*p* < 0.05).

Since copper is a redox-active metal, the dual effects of copper on oxidative stress and antioxidant activity of IPEC-J2 cells were evaluated. Intracellular ROS, MDA, GSH/GSSG, and SOD levels after incubating with different copper sources for 10 h are presented in Table 2 and Figure 3. GSH and GSSG levels determined by LC-MS/MS are listed in Table 2. GSH contents significantly decreased in all treatment groups compared to control, and GSSG contents significantly increased after 30 μM CuSO_4_, 30 μM Cu-Gly, and 120 μM CuSO_4_ incubation. The GSH/GSSG ratios were decreased significantly at both concentrations of copper as compared to the control group. Significantly different GSH/GSSG ratios were observed for organic and inorganic copper. For example, after 120 μM CuSO_4_ treatment, GSH/GSSG ratios were lowest with a 3.2-fold decrease in comparison to the control while Cu-Gly and Cu-Pro had 2.1 and 1.9-fold decreases, respectively.

Results showed a significant increase in SOD activity at 120 μM treatments compared to the control (Figure 3a). For the 30 μM group, there were no statistically significant differences in the SOD activity with different copper sources, but at 120 μM treatments, significant differences in SOD activity among the three kinds of copper were observed. Compared to control, the highest SOD activity was in Cu-Pro treated group with a 1.6-fold increase; the lowest was the CuSO_4_ treated group with a 1.2-fold increase. In addition, SOD1 protein expression levels were analyzed by Western blotting (Figure 3b). No significant difference was observed in the 30 μM CuSO_4_, 30 μM Cu-Gly, and 120 μM CuSO_4_ treatment groups compared to control. But SOD1 protein expression levels were significantly upregulated in 30 μM Cu-Pro, 120 μM Cu-Gly, and 120 μM Cu-Pro treatment groups with 1.4, 1.4, and 2.0-fold, respectively, compared to control.

Figure 3c indicates there were significant increases in the intracellular ROS generation in three 30 μM treatment groups and 120 μM CuSO_4_ treatment group, but no statistical difference was observed in 120 μM Cu-Gly and Cu-Pro treatment group compared to the control.

The MDA generation significantly increased after exposure to 30 μM copper (Figure 3d). A significant difference in MDA content between organic and inorganic copper was observed, the highest MDA content was in Cu-Pro treated group with a 2-fold increase over the control, and the lowest was the CuSO_4_ treated group with a 1.6-fold increase. Conversely, in the 120 μM treatment group, the MDA content was decreased compared to the 30 μM exposed group. The MDA content of the 120 μM CuSO_4_ treatment group was significantly higher than that of the control, Cu-Gly, and Cu-Pro groups.

### 3.4. The mRNA Expression of CTR1, ATOX1, ATP7A, ASCT2, and PepT1 Genes

**Figure 4:**
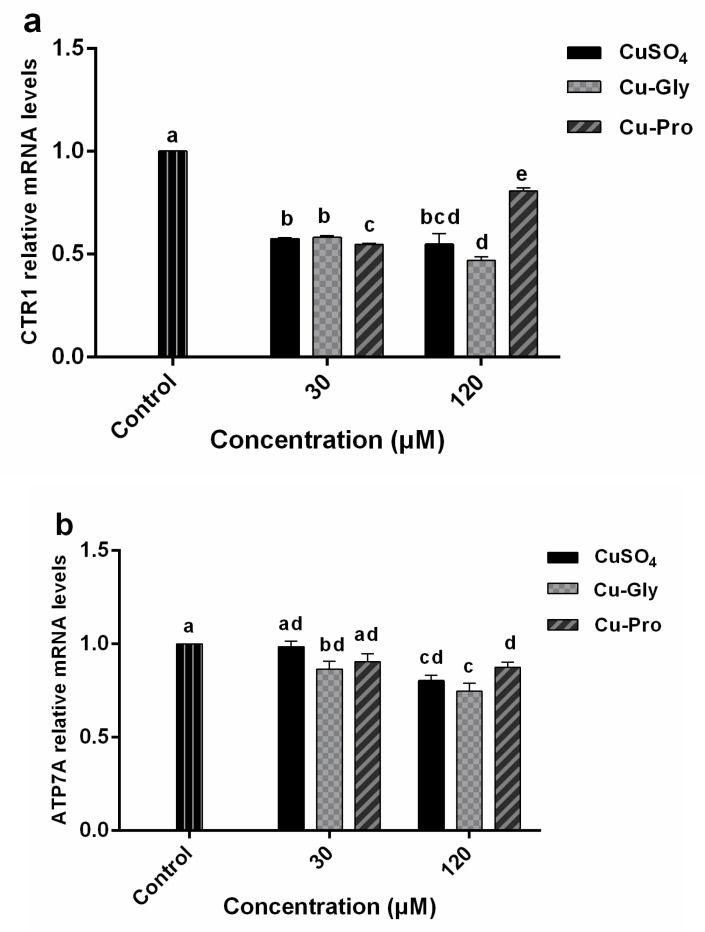

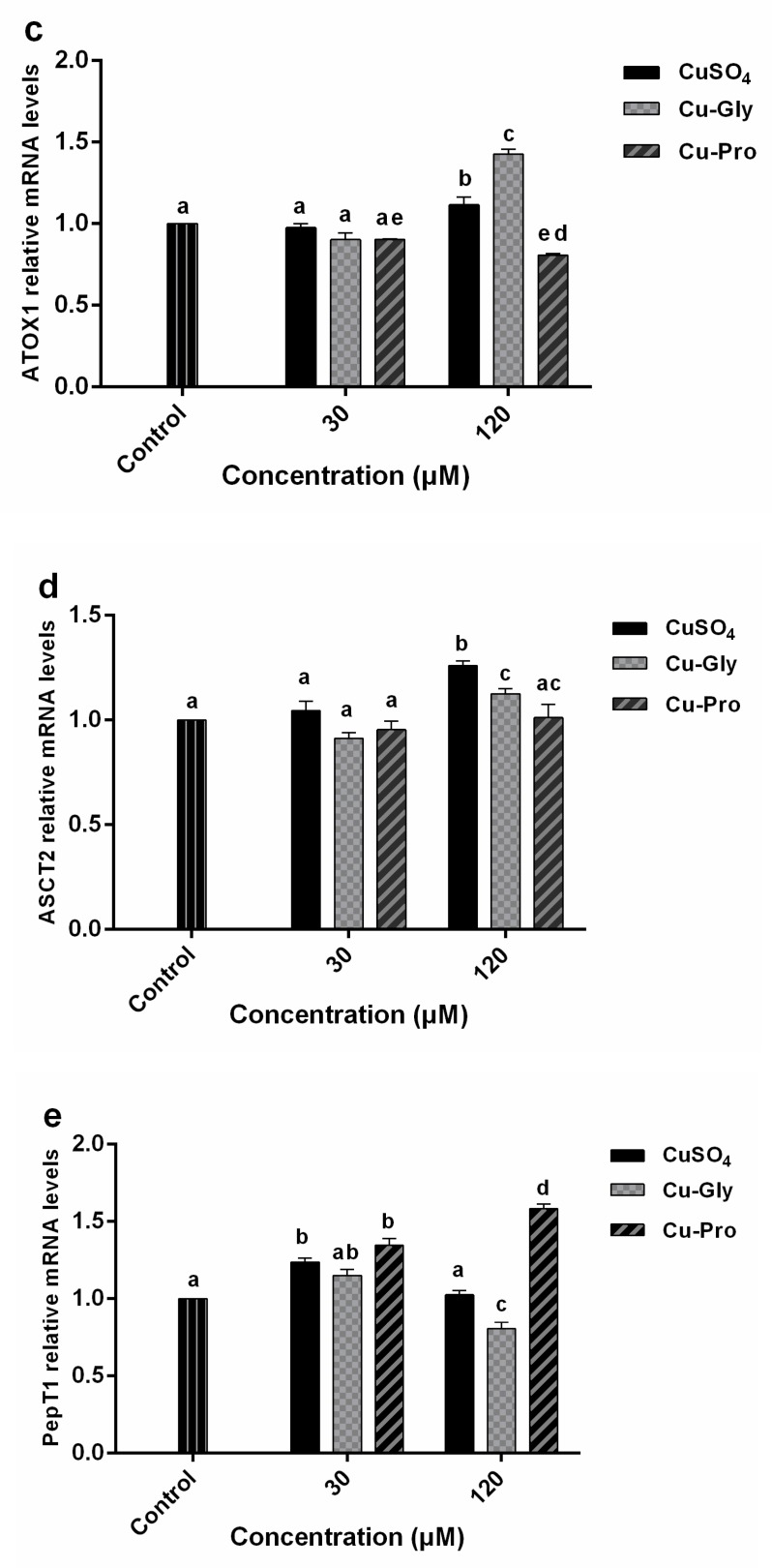
The relative mRNA expression levels of *CTR1* (**a**), *ATOX1* (**b**), *ATP7A* (**c**), *ASCT2* (**d**), and *PepT1* (**e**) in IPEC-J2 cells. Cells were treated with CuSO_4_, Cu-Gly, and Cu-Pro at the concentrations of 30 and 120 μM for 10 h. *β-actin* was used as the reference gene. The results were normalized to the gene expression in control conditions. Data represent mean values ± standard deviation (*n* = 3). Significant differences between processing groups are represented by different lowercase letters (*p* < 0.05).

Relative mRNA levels of various genes were determined in IPEC-J2 after treatment with CuSO_4_, Cu-Gly, and Cu-Pro. The results showed that a significant downregulation of *CTR1* gene expression (Figure 4a) was observed in both concentrations of copper treatments. For the 120 μM exposed group, *CTR1* gene expression levels were significantly different among three kinds of copper. Compared to the control, 1.8-fold, 2.2-fold, and 1.25-fold downregulation were observed after CuSO_4_, Cu-Gly, and Cu-Pro exposure, respectively. *ATP7A* (Figure 4b) gene expression was also significantly downregulated in the 120 μM exposed group.

A significant upregulation of *ATOX1* gene expression (Figure 4c) was observed in the presence of 120 μM CuSO_4_ and Cu-Gly with 1.1-fold and 1.4-fold increases while a 1.2-fold downregulation was observed in the presence of 120 μM Cu-Pro. For the 30 μM exposed group, there was no difference between the treatment group and the control group. *ASCT2* gene expression (Figure 4d) was significantly upregulated by 120 μM Cu-Gly and CuSO_4_ exposure with 1.3-fold and 1.4-fold, respectively. For the 30 μM exposed group, *PepT1* gene expression was significantly increased by CuSO_4_ and Cu-Pro exposure, with no difference among the three copper sources. However, *PepT1* expression (Figure 4e) was upregulated 1.6-fold after 120 μM Cu-Pro exposure and downregulated 1.3-fold after 120 μM Cu-Gly exposure.

### 3.5. The Effects of Copper on CTR1 and PepT1 Protein Expression

**Figure 5:**
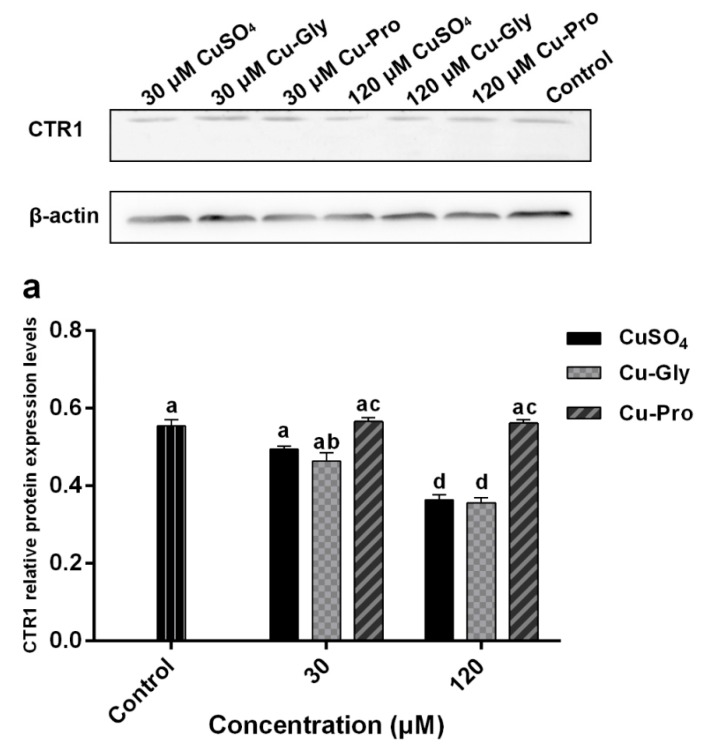

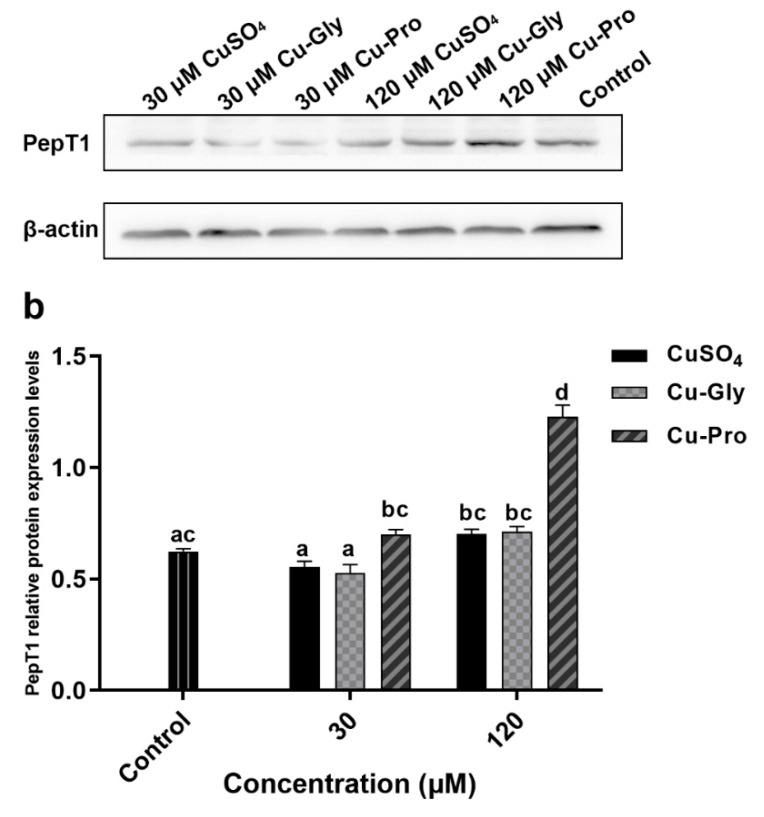
The protein expression of (**a**) CTR1 and (**b**) PepT1 measured by Western blotting. Cells were treated with CuSO_4_, Cu-Gly, and Cu-Pro at the concentrations of 30 and 120 μM for 10 h. The densitometry of blot images normalized to β-actin levels for each lane. Significant differences between processing groups are represented by different lowercase letters (*p* < 0.05).

Figure 5 shows the protein expression of CTR1 and PepT1. Compared to the control, significant downregulation of CTR1 was observed after CuSO_4_ and Cu-Gly pretreatments at 120 μM. There was no difference between the Cu-Pro treatment group and control. However, the expression of PepT1 protein was significantly upregulated by 120 μM Cu-Pro treatment group with 1.9-fold, with no difference resulting from either level of CuSO_4_ or Cu-Gly pretreatments.

## 4. Discussion

Copper is an essential trace element that has been involved in various biological processes. In this study, we mainly focused on the uptake, metabolism, and the damage induced by different copper sources and compared the expression of various genes *CTR1, ATOX1, ATP7A, ASCT2* and *PepT1* between normal and copper exposed IPEC-J2 cells.

Significantly increased copper content in cells was observed with the rising doses of three copper sources. Cellular copper contents were affected by copper supplementation. The absorption efficiency of organic copper was higher than that of inorganic copper at 120 μM level as the uptake rate of copper was Cu-Pro > Cu-Gly > CuSO_4_. Moreover, the detection results of CCK-8 assay and EdU staining assay showed that the cell viability was significantly decreased with rising copper exposure, and the cell viability after Cu-Pro incubation was significantly higher than CuSO_4_ and Cu-Gly incubation at both doses. These findings indicated that organic and inorganic copper exposure in intestinal cells could induce varying extents of cell injury.

To assess the cell injury, we also measured several biomarkers, such as GSH, SOD, ROS, and MDA, which are crucial biomarkers in the oxidative stress process. GSH and SOD act as scavengers of oxygen free radicals and protect the organism against negative effects. Under oxidative stress conditions, GSH is generally oxidized or catalyzed by glutathione peroxidase (GPX) to GSSG, and the ratio of GSH to GSSG is changed. GSH/GSSG is an important parameter indicating the extent of oxidative stress in a biological system [20]. In this study, GSH/GSSG ratios of copper treatment groups were always significantly lower than that of the control group within the rising copper concentrations. GSH, as a vital antioxidant, may be oxidized to GSSG in the oxidative stress process caused by excess Cu, resulting in GSH depletion [9,21]. The GSH/GSSG ratios of the CuSO_4_ treated group was significantly lower than that of Cu-Gly and Cu-Pro treated groups at both 30 and 120 μM, which may also indicate the cytotoxicity of organic copper and inorganic copper may be driven by a different mechanism.

Under the induction of free radicals and the compensatory stress of an organism, the cells or the organism, will inductively enhance the antioxidant capacity, and generally, SOD levels will increase. However, with the high consumption of SOD by high concentrations of free radicals and the decompensation of the organism, the biosynthesis ability of SOD will soon fall back to a low level and maintain a new dynamic balance with higher concentrations of free radicals [22,23]. In our research, there was no significant difference between control cells and the 30 μM copper exposed groups. However, the SOD activity of 120 μM copper exposed groups was significantly increased in comparison to control cells, and the levels of SOD activity were ordered as Cu-Pro > Cu-Gly > CuSO_4_, which indicated a significant antioxidant reaction occurred, and it seemed to be more easily induced by organic copper than inorganic copper. In addition, the SOD1 protein expression level was also investigated. SOD1 protein expression levels were significantly upregulated by 30 μM Cu-Pro, 120 μM Cu-Gly, and 120 μM Cu-Pro incubation. Copper is an important co-factor of SOD1; however, referring to copper uptake data, intracellular copper content was increased significantly at 30 μM treatment, but there was no significant change in SOD1 protein content except for Cu-Pro group. As the intracellular copper contents increased to a higher level, the expression of SOD1 protein increased significantly in the organic cooper treatment group compared to control. And SOD1 protein expression level in the 120 μM CuSO_4_ treatment group was significantly higher than that in the 30 μM CuSO_4_ treatment group. Intracellular copper content may not lead to an increase in SOD1 protein expression until it reaches a certain concentration in CuSO_4_ and Cu-Gly groups. In the 120 μM CuSO_4_ treatment group, SOD activity was significantly increased but not for protein expression level; it may be upregulated due to oxidative stress. In the 120 μM Cu-Gly and Cu-Pro treatment groups, SOD1 protein content was significantly increased, which may be as a result of the high accumulation of copper.

Copper, as a transition metal ion, can participate in the formation of reactive oxygen species [24]. Therefore, we measured intracellular ROS generation. The intracellular ROS generation was significantly increased after all 30 μM and 120 μM CuSO_4_ incubations compared to the control, which indicated the oxidative stress occurred in these treatment groups. But there was no significant difference in the 120 μM Cu-Gly and Cu-Pro treatment groups compared to the control. This may have been contributed to by the antioxidant effect of GSH and SOD, which caused the difference in intracellular ROS generation to not reach a significant level and maintain a balance. When an oxygen-free radical acts on lipid peroxidation, the final oxidation product is malondialdehyde, which has deleterious effects and is generally regarded as the reflex of cell injury [25]. MDA content was significantly increased in copper exposed cells compared to control cells. This result also strongly proved that oxidative stress occurred in cells. Interestingly, the MDA content of the 120 μM copper exposed groups declined in comparison with the 30 μM copper exposed groups, which probably resulted from the compensatory increase of SOD, and a new dynamic balance of oxidation-reduction was established. Consistent with the preceding intracellular ROS generation, significant lower MDA content was observed in the presence of 120 μM Cu-Gly and Cu-Pro compared to CuSO_4_. That is, the absorption efficiency of organic copper is higher than that of inorganic copper, but the cytotoxicity of organic copper is lower than that of inorganic copper. These results are consistent with previous studies: Wang et al. found that 30 to 300 μM copper exposure caused about a 20% to 60% reduction in cell viability of brain microvascular endothelial cells. And SOD activity increased with rising copper levels [26]. Yang et al. investigated the cytotoxicity of excessive copper (Cu)-induced in chicken hepatocytes. The results indicated that excessive Cu (100 μM) could increase levels of ROS generation, SOD activity, and MDA content, and decrease cell viability and GSH content [27]. In the study of Nazim Husain et al., they found after copper chloride (0.01 mM–2.5 mM) incubation, ROS production and lipid peroxidation were enhanced, GSH content was decreased in erythrocytes [28].

Copper homeostasis in mammals requires complex processes of absorption and excretion, which is accomplished by regulation of copper import, intracellular flux, and efflux across the basolateral intestinal membrane [29,30]. CTR1 is a homotrimeric protein that transports Cu across the plasma membrane with high affinity and specificity [31]. The import of copper by CTR1 does not require energy or a proton gradient, but efflux requires hydrolysis of ATP. Two well-studied ATPases are known to participate in copper homeostasis, ATP7A and ATP7B. Among them, ATP7A is expressed in the intestinal epithelium as well as in most other tissues other than the liver [32,33]. Cu is carried to Cu-dependent enzymes or subcellular compartments through the target-specific Cu chaperone proteins that include Atx1/Atox1, Cox17, and CCS. The Atx1/Atox1 Cu chaperone directly interacts with the cytosolic Cu-binding domains of the Cu transporting P-type ATPases [34].

There are studies showing that organic metals, such as proteinates, may increase mRNA expression of other transporters, such as the intestinal peptide transporter (PepT1), a high-capacity, low-affinity intestinal transporter that is expressed on the apical membrane of endocrine cells [35]. In addition, glycine can be transported by an ASC-type transporter, the important transit mechanism for neutral amino acids [36].

In our study, copper import transporter *CTR1* was downregulated in all treatment groups, and copper export transporter *ATP7A* was downregulated with 120 μM copper exposure. The results suggested that in the transcription process, cells decreased the expression of these two genes to regulate intracellular copper flux, thus responding to elevated copper concentrations, except for the Cu-Pro treatment group, because at the translational level Cu-Pro treatment group was not consistent with mRNA expression. It will be discussed later. Further, the increase of *ATOX1* expression after 120 μM copper exposure may be correlated with the significant higher intracellular copper contents after copper treatments. We also observed the significant upregulation of the *PepT1* (in 30 μM Cu-Pro, 30 μM CuSO_4_, and 120 μM Cu-Pro groups) and *ASCT2* (in 120 μM Cu-Gly and CuSO_4_ groups), which meant peptide and amino acid transport pathways were activated.

Furthermore, at the protein level, the protein expression of CTR1 and PepT1 was altered with Cu treatments. Consistent with the mRNA expression, CTR1 protein expression decreased after 120 μM CuSO_4_ and Cu-Gly exposure, it may be a response to elevated copper concentrations to prevent more copper from being transported. However, inconsistent with mRNA expression, there was no significant difference in CTR1 protein expression between Cu-Pro treatment group and control. In other words, at the protein level, the inhibitory effect of high concentration copper on CTR1 expression was not observed in the Cu-Pro treatment group. The CTR1 protein content of the Cu-Pro treatment group was higher than that of CuSO_4_ and Cu-Gly treatment groups, so it may transport more copper into cells. The results may explain why copper uptake was higher in cells treated with Cu-Pro as compared to CuSO_4_ and Cu-Gly at 120 μM. But it cannot explain the high absorption efficiency of 120 μM Cu-Gly compared to 120 μM CuSO_4_ and control. PepT1 protein expression was only upregulated after 120 μM Cu-Pro exposure, which indicated that the level of the peptide from 120 μM Cu-Pro can increase the expression of PepT1 protein. More peptides and amino acids may be transported into cells, and it may have a positive effect on cells.

Some studies have similar results to ours. Cheng et al. found that CTR1 and ATP7a mRNA levels declined in fish anterior intestine with increasing dietary Cu levels [37]. In the study of Liao et al., examining the different mechanisms of absorption of diverse iron sources in pig intestinal epithelial cells, PepT1 transcription expression in ferrous bisglycinate groups was significantly higher in treatments with 0.5 and 1 mM iron at 24 h, compared with ferrous sulfate [38].

However, there are still some limitations to our research. We cannot confirm whether PepT1 and ASCT2 are able to transport amino acids and peptides as well as copper as the causative mechanism for the higher absorption rate of organic copper. According to existing results, the high absorptivity of Cu-Pro may be due to CTR1 protein expression not being suppressed. Additionally, the reason for the difference in cytotoxicity between organic copper and inorganic copper, especially CuSO_4_ and Cu-Pro, is still unknown. Therefore, the area of differential copper transport mechanisms and cytotoxicity for various copper sources still require more in-depth research.

In conclusion, copper supplementation induced intestinal epithelial cell injury and oxidative stress in vitro. Therefore, high doses of copper supplementation in swine production should be carefully considered. Compared with inorganic copper (CuSO_4_), organic copper (Cu-Pro) was less toxic and had higher absorption efficiency at the higher dose. However, the different transport mechanisms between inorganic and organic copper need to be further investigated.

## Figures and Tables

**Table 1:** Oligonucleotide primers used for RT-qPCR.

Name	GenBank Accession No.	Primer Sequence (5′-3′)	Product Size (bp)
*β-actin*	AJ312193.1	F:GGATGCAGAAGGAGATCACG	130
		R:ATCTGCTGGAAGGTGGACAG	
*CTR1*	AF320815.2	F:CTGGACCAAATGGAACTATCC	107
		R:CTGATGACCACCTGGATGATA	
*ATOX1*	NC_010458.4	F:CCGAAGCACGAGTTCTCC	109
		R:TGTTGGGCAGGTCAATGTC	
*ASCT2*	DQ231578.1	F:CAAGATTGTGGAGATGGAGGAT	132
		R:TTGCGAGTGAAGAGGAAGTAGAT	
*PepT1*	AY180903.1	F:CCCAGGCTTGCTACCCAC	144
		R:ACCCGATGCACTTGACGA	
*ATP7A*	AY011428.1	F:GGCTGCTTCATCTGTTTCAGTA	100
		R:TTTCTGTCCCATCTGGCTT	

**Table 2:** Reduced and oxidized glutathione (GSH and GSSG) levels in IPEC-J2 cells determined by LC-MS/MS (*n* = 3). Cells were treated with CuSO_4_, Cu Glycine (Cu-Gly), and Cu proteinate (Cu-Pro) at the concentrations of 30 and 120 μM for 10 h. Significant differences between processing groups are represented by different lowercase letters (*p* < 0.05).

Copper Sources	GSH (nM/mg)	GSSG (nM/mg)	GSH/GSSG
Control	1.35 ± 0.15 ^a^	0.028 ± 0.003 ^a^	47.53 ± 0.11 ^a^
30 μM CuSO_4_	0.94 ± 0.09 ^b^	0.091 ± 0.03 ^b^	12.68 ± 1.33 ^b^
30 μM Cu-Gly	0.99 ± 0.12 ^b^	0.043 ± 0.008 ^c^	22.02 ± 0.84 ^cd^
30 μM Cu-Pro	0.72 ± 0.08 ^b^	0.038 ± 0.007 ^ac^	19.76 ± 1.08 ^c^
120 μM CuSO_4_	0.74 ± 0.08 ^b^	0.056 ± 0.011 ^cd^	14.81 ± 0.65 ^b^
120 μM Cu-Gly	0.82 ± 0.07 ^b^	0.037 ± 0.006 ^ac^	22.05 ± 1.33 ^c^
120 μM Cu-Pro	0.84 ± 0.05 ^b^	0.032 ± 0.004 ^ac^	24.63 ± 0.46 ^d^
